# Transitioning to a virtual post-intensive care rehabilitation service in response to the COVID-19 pandemic: results of multidisciplinary focus-groups

**DOI:** 10.3389/fmed.2024.1513121

**Published:** 2025-01-03

**Authors:** Fiona Howroyd, Natacha Earle, Jonathan Weblin, David McWilliams, Mark Raven, Niharika A. Duggal, Zubair Ahmed, Tonny Veenith

**Affiliations:** ^1^University Hospitals Birmingham NHS Foundation Trust, Queen Elizabeth Hospital Birmingham, Birmingham, United Kingdom; ^2^Department of Inflammation and Ageing, School of Infection, Inflammation and Immunology, College of Medicine and Health, University of Birmingham, Birmingham, United Kingdom; ^3^Centre for Care Excellence, University Hospitals Coventry and Warwickshire NHS Trust, Coventry, United Kingdom; ^4^Royal Wolverhampton Hospital, New Cross Hospital, Wolverhampton, United Kingdom

**Keywords:** telehealth, telerehabilitation, physiotherapy, physical therapy, SARS-CoV-2, critical care

## Abstract

**Background:**

Telehealth has vastly expanded since the SARS-CoV-2 (COVID-19) pandemic and has been widely implemented as an efficient, cost-effective and accepted means of health care delivery, including rehabilitation. Although telerehabilitation is recommended across national guidelines, there is a lack of practical guidance to support clinicians with virtual adaptations.

**Aims:**

This study aimed to describe the key components of a safe and effective virtual post-intensive-care rehabilitation service, through qualitative exploration.

**Methods:**

This is a qualitative study using a focus-group design based upon grounded theory. This study is nested within a service development project, taking place during the COVID-19 pandemic. Focus groups were held after the first wave of the COVID-19 pandemic with key stakeholders from the physiotherapy and critical care departments of a large tertiary hospital in the United Kingdom. Semi-structured questions were used to guide discussions, led by a facilitator and scribe. Transcripts were thematically analysed using an exploratory inductive approach by two researchers then crosschecked.

**Findings:**

Three focus groups were attended by 12 multidisciplinary stakeholders, including six physiotherapists, two administration staff members, two critical-care follow-up nurses and two critical care consultants. Thematic analysis identified seven critical elements for virtual adaptations: (1) safety and risk assessment, (2) assessment and outcome measures, (3) virtual platform, (4) resources and equipment, (5) exercise programme adaptation, (6) exercise monitoring and safety, and (7) privacy and information governance.

**Conclusion:**

Our findings provide practical recommendations for virtual rehabilitation service development and delivery.

## 1 Introduction

Telehealth can be defined as the use of telecommunications technology for health care delivery, monitoring and education (1). Originally, telehealth provided access to essential healthcare to under-represented and disadvantaged populations or those in rural areas (2). However, telehealth has vastly expanded since the SARS-CoV-2 (COVID-19) pandemic and has been widely implemented as an efficient, cost-effective and accepted means of health care delivery, including rehabilitation (3–8). Telerehabilitation is as effective as face-to-face therapy in multiple sectors (4, 9). Specifically, recent meta-analyses have reported comparable outcomes for virtual and face-to-face rehabilitation, including improvements in: pain and physical function in patients with musculoskeletal conditions and following orthopaedic surgery (10, 11), activities of daily living after stroke (12), exercise capacity for cardiac conditions (13) and a reduction in acute exacerbations and hospital admissions for chronic obstructive pulmonary disease (14). National guidelines have since advocated the evolution of remote services and virtually delivered rehabilitation (15–17).

The critical illness induced by COVID-19 resulted in a vastly growing cohort of intensive care unit (ICU) survivors, with up to 80% of patients requiring ongoing care rehabilitation after hospital discharge (18–21). The rising demand for post-intensive-care follow-up presented significant challenges to rehabilitation services in an area that was already nationally scarce and under-resourced (16, 22–26). This pressure was further complicated by the strict infection control and social distancing regulations, repurposing of rehabilitation spaces, and redeployment of staff to work in frontline services during the pandemic (23).

As more patients survive critical illness, there is an ever-increasing requirement to prioritise follow-up care and optimally utilise resources for patients suffering from long-term multimorbidity propagated by critical illness (18, 27). Post-Intensive Care Syndrome (PICS) describes the long-lasting effects of critical illness. It encompasses physical, cognitive, and psychological symptoms that affect at least half of all ICU survivors (18, 28). PICS is recognised as a public health burden due to its lasting functional disability and the risk of persistent physical and non-physical morbidity (24). The management of PICS requires ongoing rehabilitation support for patients discharged after a critical illness, with post-intensive-care follow-up recommended by UK national guidance (26).

As we emerge from the pandemic, there continues to be an overwhelming demand for healthcare services, with ever-increasing waiting lists, cost pressures and service restructuring. Therapists are also faced with the ongoing loss of space and facilities, as many rehabilitation departments have faced permanent repurposing beyond the pandemic (29). However, for decades before the pandemic, access to rehabilitation has been poor, with under-commissioned and fragmented services (30). Despite national guidance and recommendations, there is a global shortage of publicly funded rehabilitation, with patients often waiting weeks to months for vital therapy services (25, 26, 30–33).

Although national guidelines now advocate telehealth, there is a lack of robust evidence and structured guidance to support clinicians practically when developing telerehabilitation services. There is a need for practical advice that can empower clinicians and key stakeholders to prioritise and implement virtual post-ICU follow-up services. The article refers to the processes that led to a successful virtual post-intensive-care rehabilitation service at a quaternary referral centre in the United Kingdom (UK) (34).

The aim of this present study was to describe the key components for safe and effective post-intensive-care rehabilitation during its transition to a virtually delivered service, operating through the COVID-19 pandemic, by qualitative exploration.

## 2 Materials and methods

### 2.1 Study design

This is a qualitative study using focus-group design based upon grounded theory. This study is nested within a service development project, taking place during the COVID-19 pandemic.

### 2.2 Research setting

The virtual service was developed in a single-centre, major tertiary hospital in the United Kingdom during the first wave of the COVID-19 pandemic (34). Prior to the pandemic, the critical care’s physiotherapy department offered weekly, in-person, post-intensive-care rehabilitation classes for survivors of critical illness. The class consisted of structured group exercise classes led by ICU physiotherapists in the hospital’s therapy outpatient department gymnasium, followed by support groups led by the critical care nurse follow-up team. This outpatient-based physical rehabilitation programme was a well-established, safe and effective service, with previously published benefits upon physical and psychological outcomes (35–38). However, due to the demands of the COVID-19 pandemic, all outpatient services were suspended to repurpose staff and resources to frontline emergency services. As one of the largest single-site critical care units in the United Kingdom, the hospital expanded its capacity by 500% for the vast influx of critically ill adults (39). Although this provided essential life-saving care in the acute stages of illness, it was soon recognised that survivors were facing extensive neuromuscular weakness and a loss of wellbeing, with significant rehabilitation needs beyond ICU and hospital discharge (20).

### 2.3 Procedure

After the first wave of the COVID-19 pandemic, we set up a multidisciplinary working group to discuss the development of a virtual post-intensive-care rehabilitation service. Participants were recruited by opportunistic convenience sampling involving key stakeholders in the hospital with clinical expertise in outpatient rehabilitation and post-intensive-care service. The inclusion criteria consisted of staff with experience of outpatient and post-intensive-care rehabilitation and exclusion was refusal to participate. Participants were approached verbally by physiotherapists who led the post-intensive-care rehabilitation service, inviting staff members to engage in a service development project. The aim of the project was to adapt the previous face-to-face post-intensive-care rehabilitation service to a virtual platform during the COVID-19 pandemic. Participation was voluntary, yet as this study was nested within a service development project, all stakeholders were readily available as part of routine clinical services and thus the team were known to one-another. Participants were approached face-to-face in the hospital setting.

We explored the main issues and challenges of delivering a virtual rehabilitation programme and identified ways of developing a safe virtual service. Semi-structured questions (Table 1) were established using the healthcare complex interventions framework to encourage a dynamic, problem solving approach with clinical relevance (40). Each focus group ran for approximately 60 min, taking place in-person in meeting rooms in the hospital, abiding to social distancing rules. The meetings were attended by a facilitator and scribe (FH) who was also a member of staff directly involved in the virtual service development to provide detailed interrogation of clinically relevant topics. The facilitator (FH) was a female and critical care physiotherapist with clinical research experience with previous qualitative research training, who was known by all members of the focus-group as a colleague and team member. All notes were written down to maintain the integrity of the issues raised and discussed.

**Table 1:** Semi-structured questions for focus-group meetings.

Question (prompts in *italics*)	
1	What would a virtually delivered post-intensive-care service look like? *What, when, where, how, who?*
2	Is a virtual service adaptation feasible? *Logistics, processes, equipment, governance, IT services*
3	Is a virtual service appropriate? *How can we optimise patient safety, engagement and effectiveness*
4	How can we measure effectiveness? *Outcome measures*

### 2.4 Data analysis

Two clinical physiotherapists (FH and DM) with experience in conducting qualitative research interrogated the notes and manually organised the transcripts by hand prior to thematic analysis. The transcripts were combined together for analysis and thus between group comparisons were not performed. Participants were assigned an individual identification number and thus the job role and background of each participant was not included within sub-analysis. After familiarisation with the transcripts, data were initially labelled with codes that identified commonly occurring themes including safety, patient factors and logistical/service-related factors. The transcripts were thematically analysed using an exploratory inductive and process-driven approach to organise major themes and sub-themes, then crosschecked for consistency (41). The focus groups followed the criteria of the Consolidated Criteria for Reporting of Qualitative Research (COREQ) checklist for interviews and focus groups (42) (Supplementary File S1).

### 2.5 Ethical considerations

This service evaluation was registered with the Clinical Audit and Registration Management System (CARMS) (CARMS Identification Number: CARMS-17954). No identifiable data or patient information was used for this study or manuscript development and hence this work did not require ethical approval. This work formed part of a service development project and was an adaptation to existing clinical care services.

## 3 Results

Twelve stakeholders were approached and agreed to participate. The stakeholders consisted of: critical care physiotherapists (*n* = 2), musculoskeletal outpatient’s physiotherapists (*n* = 3), outpatient department lead physiotherapist (*n* = 1), administration staff (*n* = 2), critical-care follow-up nurses (*n* = 2) and critical care medical consultants (*n* = 2). The group included 9 females and 3 males, all were adults between the age of 30 and 60, each with at least 10 years of experience working in the acute hospital setting. Two staff members were critical care specialist physiotherapists with previous experience of post-intensive-care rehabilitation care in addition to acute critical care experience during the COVID-19 pandemic and so were aware of potential issues that may arise with this patient cohort and the potential challenges of virtual adaptation. One of the critical care physiotherapists was a consultant lead and had established links and networks with national and international critical care physiotherapists. The clinical lead for the physiotherapy outpatient department had operational and managerial oversight of local policies and procedures linked to risk assessment, information governance and the Trust’s virtual transition plans, with links to the Trust’s information technology and communications teams. The stakeholders participated in three focus groups based upon individual availability. Each focus group consisted of 4 to 5 people, in order to maintain appropriate social-distancing due to the infection-control procedures during the COVID-19 pandemic. There were no drop outs during the period of the service development process.

All stakeholders were supportive of the transition from face-to-face to virtual rehabilitation.

P1: “*virtual follow-up would provide a COVID friendly alternative*.”

P2: “*we have a huge cohort of ICU survivors with extensive rehab needs, virtual rehab is the only option, we have to adapt.”*

However, as this cohort had been recently critically unwell, with little known about the lasting effects of COVID-19 at the time of service development, maintaining patient safety was at the forefront of all discussions.

P2: “*we do not yet know the lasting effects of COVID, how do we know if exercise is safe, especially if delivered remotely without monitoring*.”

P6: “*we need to protect staff and patients from COVID and maintain social distancing… virtual is a great alternative, but is it safe?”*

**Figure 1:**
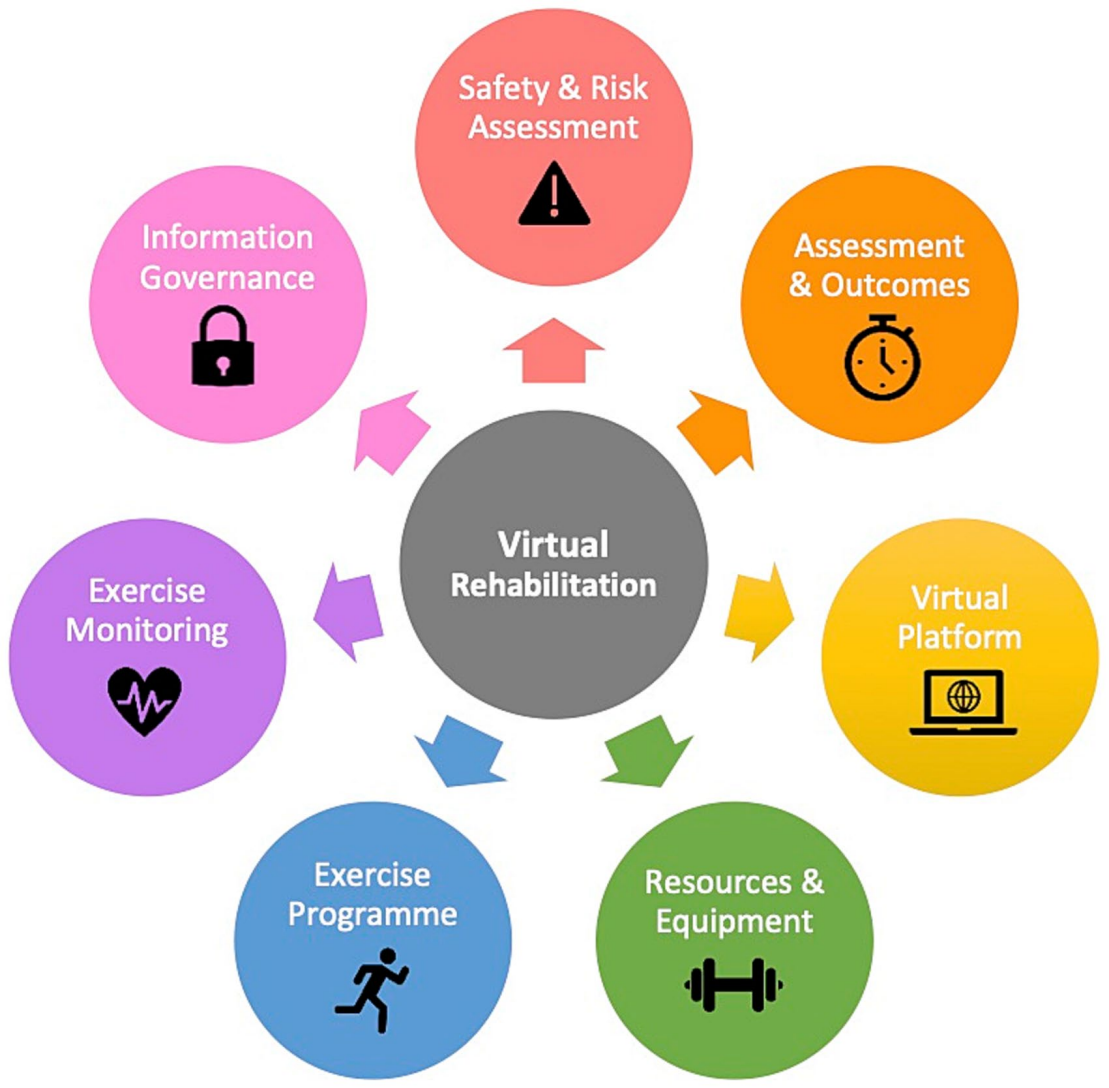
Seven critical themes for developing a virtual rehabilitation service.

Thematic analysis identified seven critical elements for virtual adaptations: (1) safety and risk assessment, (2) assessment and outcome measures, (3) virtual platform, (4) resources and equipment, (5) exercise programme adaptation, (6) exercise monitoring and safety, and (7) privacy and information governance (Figure 1). Further analysis of Theme 2 identified three sub-themes; (2a) pre-assessment, (2b) outcome measures, (2c) patient selection.

Based on the seven key themes and following the successful implementation of our local service evaluation (34), recommendations for virtual rehabilitation adaptations have been detailed below. The quotes demonstrate some of the early concerns and queries raised by members of the group during the initial stages of virtual rehabilitation development. The text then describes how these concerns were overcome and what processes were put in place for successful service development, with practical advice and post-implementation reflection.

### 3.1 Theme 1: safety and risk assessment

During the early stages of virtual transitioning, safety was the primary concern and focus for all elements of service development.

P6: “*But is it safe?*”

P10: “*The risk of leaving patients without rehabilitation and support has to outweigh the risk of virtual rehab, if we have the right processes in place, we can minimise risk and keep patients safe*.”

P12: “*Risk assessment is key… if patients understand the risks and benefits, they can make an informed choice*.”

Rigorous processes were therefore put in place, with established safety and risk assessment procedures. During the service evaluation period, no safety or adverse events were reported. Recommendations are detailed below.

**Figure 2:**
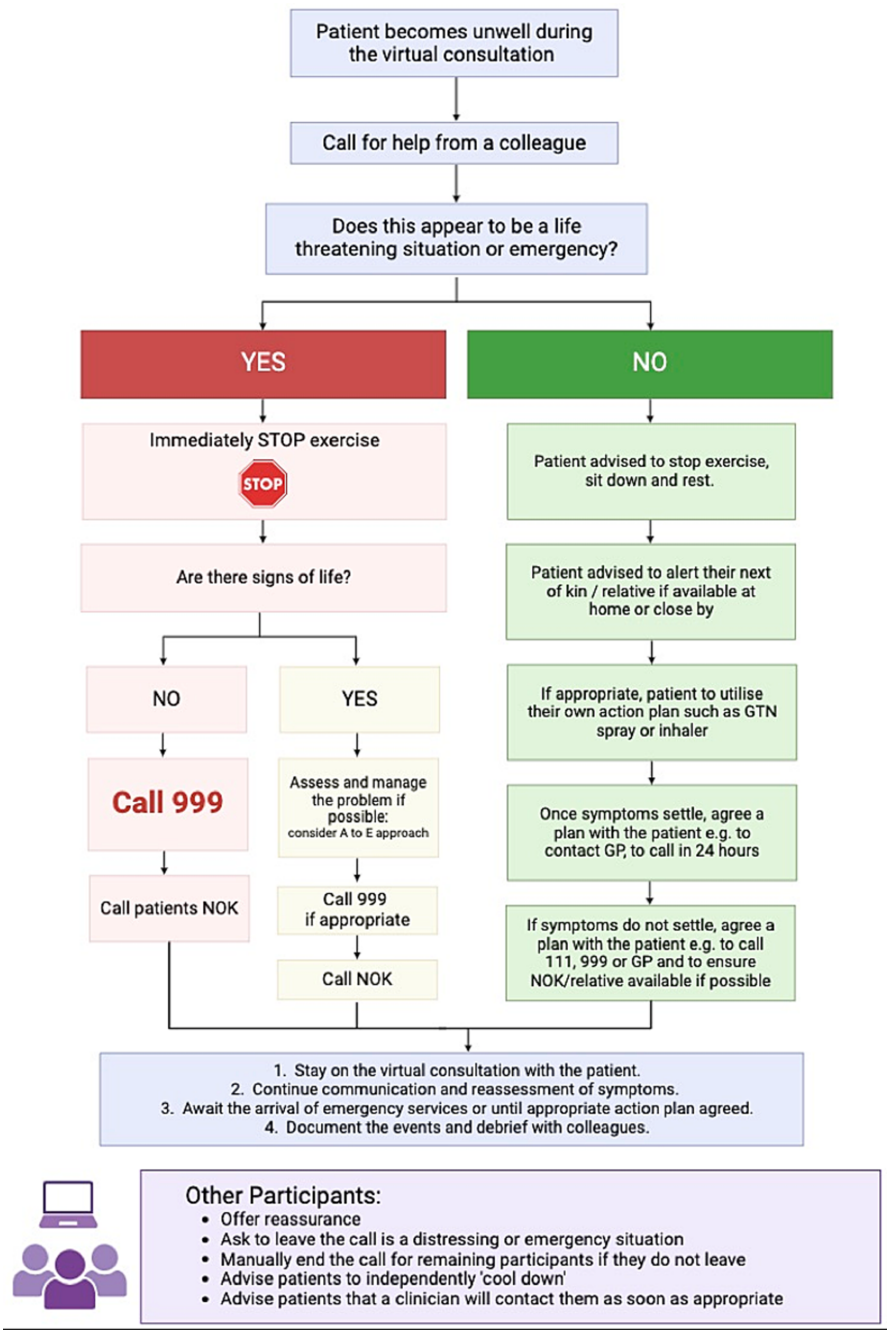
Emergency flow diagram in case of a medical event during virtual rehabilitation. NOK, next of kin; GP, General practitioner.

A thorough risk assessment is paramount to a safe virtual rehabilitation service. This should include an emergency action plan, in the event that a patient was to become unwell during virtual rehabilitation (Figure 2). In the event that a patient appears to be in a life-threatening situation or emergency, the exercise class should be immediately stopped and the emergency services must be contacted as quickly as possible and directed to the patient’s home address. The response to all other potential scenarios, where there is not an immediate risk of death or danger should be assessed on an individual case basis. All staff should be familiar with this action plan.

It should not be necessary to mandate that someone is physically present with the patient as they complete virtual rehabilitation. However, the therapist must ensure they have the correct location address of the patient and the contact details of the patient’s next of kin, for in the event of an emergency. Although only one therapist is required to deliver the rehabilitation class, a second staff member should be near to assist with the action plan in an emergency. In this scenario, the second staff member would then be able to make any required phone-calls, such as to emergency services or the patients next of kin, whilst the class therapist remained on the video call with the patient. The patient’s medical records, including their address and next of kin phone number, would be on-hand during the class, with a phone-line close-by. To minimise the risks of a medical emergency, there should be careful consideration for pre-assessment, the format of rehabilitation delivery and patient monitoring, as detailed in the themes below.

Patients must be informed of an appropriate ‘disclaimer’ before any virtually delivered exercise so they know that the training is completed in their own homes, and at their own risk. Advice and information should be provided verbally and in writing as listed:

- When it is not appropriate to complete the exercise for example, if the patient has a new injury or acute unstable medical condition.
- Stop exercise if the patient becomes unwell, emphasising symptoms such as chest pain, dizziness, extreme shortness of breath or acute pain.
- Ensure the patient has appropriate space to exercise, minimise trip or fall hazards and ensure suitable footwear and clothing is worn.
- Ensure appropriate nutrition and hydration before and during the exercise.
- To inform the class therapist if the place where the patient is completing the class is different from their home address, so they know their correct location in the event of an emergency.

### 3.2 Theme 2: patient assessment and outcomes

To ensure that patients were deemed safe and appropriate to exercise, the pre-class assessment was deemed essential yet required careful planning during virtual adaptation. Three sub-themes emerged from Theme 2; (a) pre-assessment, (b) outcome measures, (c) patient selection.

#### 3.2.1 Subtheme 2a: pre-assessment

The pre-assessment process was carefully developed to ensure that patient safety and suitability for virtual rehabilitation could be appropriately assessed in a remote setting.

P1: “*We’ve seen a lot of COVID patients with cardiac instability, embolisms and high oxygen demands in the ICU. Pre-assessment needs to thoroughly check medical history and stability prior to exercising at home and virtually*.”

During the service evaluation period, no safety or adverse events were reported. However, patients were occasionally recommended to seek medical advice prior to starting rehabilitation if they reported new, undiagnosed or acutely unstable medical complaints. Furthermore, if patients were awaiting any investigations for medical complaints, they were recommended to await medical consultation prior to virtual rehabilitation. Recommendations are detailed below.

Conducting a one-to-one virtual consultation with each patient before physical activity is essential. This allows the clinician to check that the patient can confidently access the virtual platform, offering telephone support and navigation if required. Once the patient is online, the initial consultation allows the clinician to build a rapport and assess the patient’s safety and appropriateness to exercise. For our virtual service, this initial assessment was completed by a senior physiotherapist with experience in acute and subacute rehabilitation after critical illness.

A detailed patient history and a thorough review of the patient’s medical notes should be obtained before the virtually-delivered exercise. This should include:

- Complete medical history. How do the patient’s medical problems impact them? Do they suffer from pain or breathlessness?
- Are there any unstable medical conditions or conditions awaiting investigations or treatment?
- Obtain an entire drug history. Specifically, ask about anticoagulants, pain management, glyceryl trinitrate spray, inhalers, home oxygen, and nebulisers.
- Falls history or any balance or mobility issues.

A patient’s medical history requires careful consideration before enrolment in virtual exercise. Although inclusivity is fostered by offering varying levels of exercise intensity, patient safety is of the utmost priority. In rare cases, exercise is not deemed safe or appropriate in a virtual group setting. Therefore, enrolment is contraindicated, for example, if the patient suffers from unexplained or uncontrolled seizures or cardiovascular instability. In these cases, the patient is recommended to seek medical help before virtually delivering rehabilitation. Screening tools such as the Physical Activity Readiness Questionnaire can aid clinical decision-making when assessing the safety and suitability of participation in exercise (43). The pre-assessment should be documented thoroughly in the patient’s medical records, including past medical history, drug history, details of their ICU admission and any planned medical follow-up.

#### 3.2.2 Subtheme 2b: outcome measures

In order to measure the effectiveness of virtual rehabilitation, outcome measures were modified due to the remote nature of the assessment process.

P4: “*How can we assess if virtual rehab is effective? Our physiotherapy assessments normally involve hands-on physical examination.”*

During the service evaluation period, physical outcomes included the one-minute sit-to-stand test and questionnaires to measure self-reported breathlessness and upper limb function. Non-physical outcomes included the questionnaires for psychological distress, anxiety and depression. Recommendations are detailed below.

The chosen outcome measures may also require consideration. For example, a sit-to-stand test rather than a 6-min walk test can be used to assess cardiovascular fitness, or questionnaires that can be emailed. Although not necessarily validated for ICU populations, these outcome measures offer a pragmatic alternative during our virtual rehabilitation assessment. Hence, the patient remains visible on the screen, and the clinician can monitor the patient throughout the evaluation.

#### 3.2.3 Subtheme 2c: patient selection

Virtual adaptation was considered an essential alternative amidst the pandemic, yet staff had concerns about the inclusivity of remote rehabilitation.

P4: “*Is virtual appropriate for everyone? What about patients who have language or communication barriers?”*

During the service evaluation period just under a fifth of those who declined participation in the virtual rehabilitation service were due to language barrier. However, some patients did attend with a relative who could offer language interpretation. Recommendation of patient considerations and selection are detailed below.

Patients with complex needs and vulnerabilities also require careful consideration during pre-assessment. For example, some patients with communication impairments, language barrier, learning difficulties or mental illness may prefer face-to-face rather than virtual consultation (44, 45). Where possible, patients should be provided with a choice of alternative services when virtual or group-based rehabilitation does not meet specific needs or individual preferences (46). The patient group’s socio-economic and cultural needs should be carefully considered, ensuring that healthcare remains accessible and does not discriminate between different groups of patients based upon language or access to technology.

If the patient is deemed safe and appropriate to participate, the initial assessment may include time to demonstrate and practice the exercise programme one-on-one. This allows the therapist to assess the patient’s response to exercise and determine the patient’s appropriate level of exercise in a closely supervised manner before the group setting. Furthermore, this allows the therapist to provide support and encouragement alongside education on the exercise technique. This offers the patient reassurance and confidence before starting the group class, which may, in turn, improve adherence and self-efficacy.

### 3.3 Theme 3: establishing an appropriate virtual platform

Prior to the pandemic, all physiotherapy outpatient and post-intensive-care follow-up appointments took place face-to-face during in-person clinics. The group therefore had to explore the most appropriate virtual system and work alongside administrative and operational departments in the Trust during the development of the virtual service.

P1: “*We need to get the technology right – something that is free, IT secure, but easy to use, not all patients will be confident using technology*.”

Recommendations for establishing an appropriate virtual platform are detailed below.

An appropriate virtual platform is required to establish a virtual service. Although many systems (such as Zoom or Microsoft Teams) have become readily available since the pandemic, it is recommended that a system licensed by the hospital is to be used and deemed appropriate for security and governance for patient use. Notably, the system utilised should be accessible for free by the patient at the point of use.

The platform’s usability also requires careful consideration, particularly for patients with limited confidence or experience with technology. Ideally, the platform allows the patient to open a consultation with one simple ‘click here’ link rather than needing to negotiate or download specific apps. The system should also provide a secure link for each meeting so that the virtual ‘room’ can only be entered by patients who have been invited to do so. Lastly, the time limit of each call should also be considered to prevent issues with the call ending abruptly midway during a consultation.

### 3.4 Theme 4: resources and equipment

As the pre-pandemic post-intensive-care rehabilitation class took place in-person, in an outpatient physiotherapy gymnasium, the resources and equipment needed for the class also needed to adapt.

P2: “*We cannot bring people into the gym space, it would be too risky from an infection control and social distancing standpoint – remote rehab would overcome that problem, but we cannot expect patients to buy equipment*.”

P7: “*What about patient’s who cannot access remote rehab? Are we discriminating?”*

Staff did not feel it was appropriate for patients to need to buy specialist equipment, however technology access was essential for remote rehabilitation. The only resource requirement was therefore for patients to have access to smartphone, tablet device, laptop or computer with a camera and microphone to allow a video-call via Microsoft Teams. During our service evaluation period, of the 76 eligible critical care survivors invited to our virtual rehabilitation service, 11 patients declined due to lack of technology access, and there was one drop-out due to technology difficulties. Recommendations for resources and equipment are detailed below.

A list of the resources and equipment required for virtual and face-to-face post-intensive-care rehabilitation can be found in Table 2. Compared to face-to-face rehabilitation, the space and equipment needed for virtual rehabilitation are minimal. However, there must be appropriate access or investment in technology, including a stable internet connection, a secure email address, a computer with a video camera, appropriate acoustics and a screen large enough to ensure all group members are visible. According to the latest Office of National Statistics figures, the UK boasts internet access for 96% of its inhabitants (47). Thus, an internet connection is expected to facilitate telehealth for most of the UK population. Virtual rehabilitation does not require ample gym space however, it is important to consider information governance when planning an appropriate room to maintain patient privacy.

**Table 2:** Resources and equipment required for face-to-face and virtually delivered post-intensive care rehabilitation, comparing our service pre- and post-pandemic.

Component	Face to face rehabilitation	Virtual rehabilitation
	Pre-pandemic	Post-pandemic
Method	Face-to-face in a group.	Virtually in a group via Microsoft Teams.
Staff	Two physiotherapists are needed for the delivery of class and all administration, including appointment booking. The support group component needs two to three critical care follow-up nurses. Administration and reception staff were needed for appointment booking, greeting patients on arrival and taking telephone enquiries.	One physiotherapist is needed for the delivery of class and all administration, including appointment booking. Another qualified member of staff nearby in case of an emergency. The support group component needs one to two critical care follow-up nurses.
Location	For the exercise class, a hospital-based gym in the physiotherapy outpatient department of an acute hospital. For the support group, a seminar room should be large enough for 20 people. Patients and their relatives can attend.	Staff: a room large enough for four people, anywhere in the hospital, with a phone line and Wi-Fi access. Patients: attend virtually from their homes with relatives present if they wish.
Group size	Up to 10 patients per class. Relatives can attend.	Up to 6 patients per class, so all are visible on screen. Relatives can attend.
Equipment	A fully equipped gym/rehabilitation space with two static exercise bikes, two treadmills, six sets of hand-held weights and balls of different weights/sizes, a minimum of 12 chairs, a telephone, resuscitation equipment, observation monitors, cleaning equipment and water/drinks facilities.	Staff: Telephone line, computer, screen, four chairs, one ball and two weights for demonstration. Patients: smartphone, tablet, PC or laptop with a video camera, Wi-Fi and an email account. A chair and household objects such as tins instead of gym weights.
Preparation time	15 min for set-up of the gym space ready for the exercise class with all appropriate equipment	Five minutes to log in to a computer and set up camera, screen and microphone appropriately.
Class duration	60 min exercise class Up to 45 min for the support group	90 min Microsoft Teams call
Patient cost	Transport/parking costs	Nil

Patients did not require specialist exercise equipment to complete our virtual post-intensive-care rehabilitation programme, instead patients were encouraged to use household items as a substitute for weights. However, virtual rehabilitation does require the patient to access appropriate hardware, such as a smartphone, personal computer (PC), laptop or tablet device with a camera function, Wi-Fi and an email address. This raises concerns regarding technology-related healthcare discrimination.

From the patient’s perspective, virtual rehabilitation can be accessed from their home, minimising travel time or costs. During the pandemic, this was particularly beneficial during periods of social isolation, when gym and exercise facilities were closed and clinically vulnerable patients were concerned about entering the acute hospital setting. This is also beneficial for large tertiary centres that cover a large catchment area, negating geographical barriers to rehabilitation attendance and accessibility. Previous trials of face-to-face rehabilitation interventions for survivors of critical illness have encountered challenges with patient enrolment and engagement (48–50). Virtual services may offer a pragmatic and accessible solution for rehabilitation delivery, yet further research is necessary.

Although most virtual platforms can enable large or unlimited group sizes on the call, we reduced our group size to six patients so that all participants could remain visible on the screen. Although smaller group sizes may subsequently increase waiting list times, this allowed constant visibility to monitor patient safety during the class and helped maintain the fluidity of virtual group discussion and patient-therapist communication. For the delivery of the exercise class, staffing requirements are very similar (one staff member to 5 patients for face-to-face and one staff member to 6 patients for virtual). However, it is essential to note that both services require additional time for administrative duties such as contacting patients, appointment booking and documentation. Record keeping after each patient contact is necessary to keep up with local information governance policy. Administrative time for emailing the virtual ‘link’ each week is also required for virtual services. If possible, the support of administrative staff is hugely beneficial.

### 3.5 Theme 5: adaptation of the exercise programme

The exercise component of the post-intensive-care rehabilitation service also needed to adapt so the class was able to take place in patient’s homes, rather than in a gymnasium. Staff remained focused upon safety but also wanted to ensure that the remote rehabilitation class was an effective means of exercise.

P11: “*The class itself will have to change too. We need patients to stay visible on screen so we can monitor them and check they are OK, in case anything happens*.”

P2: “*But we also need to make sure it’s effective. Even though staying visible on screen, we need to make sure the exercise intensity can be tailored to the patient with an aim to progress*.”

Our service evaluation demonstrated a significant improvement in all physical and non-physical outcome measures after the 8-week virtual programme. Recommendations are detailed below.

In addition to the changes to equipment, space, and delivery method, the exercise programme may also need to adapt to virtual delivery so patients can remain visible on screen. Our virtual post-intensive-care rehabilitation class consisted of 10 exercises based around a chair. This allowed patients to stay relatively central on the screen yet also allowed the patient to grade their level of exercise independently, holding onto the chair for balance or sitting down at any time needed. The class therapist explained and demonstrated each exercise, with written patient information provided ahead of the programme (example given in Supplementary File S2). The patient self-paced each exercise’s level, speed and intensity based on their levels of perceived breathlessness (detailed in theme 6). Our virtual exercise programme consisted of four levels of exercise which the patient could aim to progress through as they continued with the 8-week programme. As the patient could select any exercise level during the class, heterogenous patient groups of differing abilities were able to simultaneously attend each session. This has motivational benefits for new class members, who can observe the progress of patients who have been attending for longer periods, whilst also providing those who are near to the end of the programme with confidence and reassurance.

- Level 1: seated programme with the patient sitting in a chair throughout
- Level 2: stood up, but holding onto a chair throughout for balance and support
- Level 3: stood up, not holding on to the chair, introduction of small weights
- Level 4: the patient is stood, remaining active throughout and using weights

The class therapist would observe the patient during the exercise class to monitor for signs of exercise effort, such as their work of breathing, colour or appearances of fatigue. Patient levels of perceived breathlessness were also recorded during and after the class (see theme 6) to help determine if the patient was exercising at the correct level of intensity. Furthermore, the class therapist would collate patient feedback at the start of each class regarding how they felt in the days following the previous class, as well as how they felt during the class to gain a depth of understanding regarding the patient’s response to exercise. The exercise level would be titrated accordingly. Although the therapist would direct and advise the patient, the aim was for the patient to be able to independently self-adjust and recognise their own response to exercise, to build confidence for self-management beyond the 8-week programme. The class therapist would adapt their communication style and advice according to patient needs, being as motivating, encouraging, reassuring or direct as indicated to maintain patient engagement. The class therapist would maintain a constant dialogue with the group during the class. At the end of each class, notes would be recorded in the patient’s medical records regarding the patients exercise level, self-reported patient feedback and their observed response to exercise.

### 3.6 Theme 6: exercise monitoring and safety

With safety at the forefront of all components of virtual development, staff were concerned about patient monitoring during remote exercise.

P1: “*We normally monitor patient obs during the class, heart rate and sats, so we can check in and monitor their response to exercise. We cannot expect patients to buy expensive monitoring equipment*.”

P4: “*But we also need to know they are working hard and gauging the right intensity*.”

Patient monitoring during exercise was adapted to ensure continuous observation by the class therapist on screen, use of patient self-reported exertion scales and the provision of patient education and advice. The results of our service evaluation showed that the class was safe and effective, with measures of physical fitness and perceived breathlessness on exertion improving after the 8-week programme. Recommendations are detailed below.

**Figure 3:**
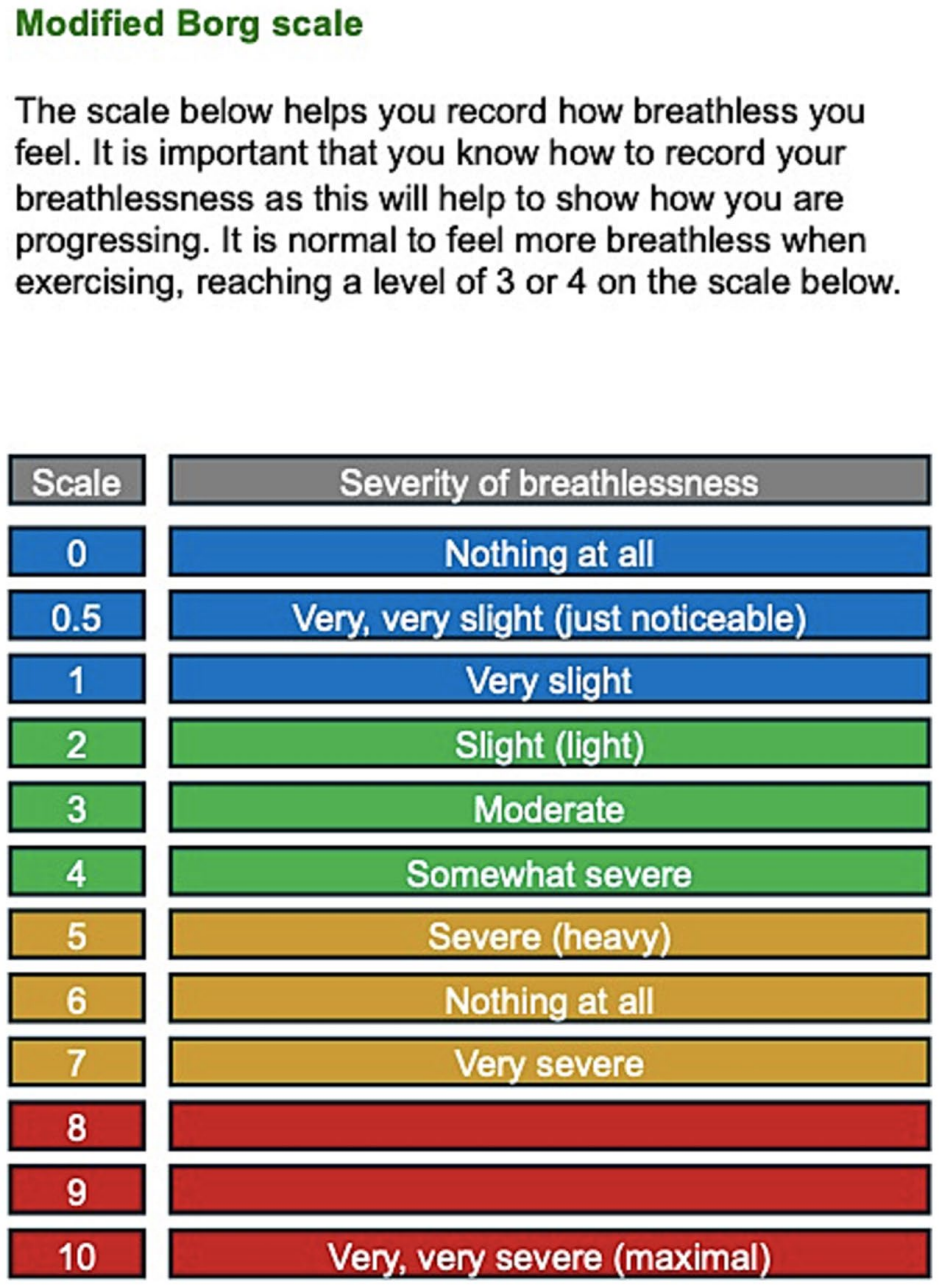
Modified Borg scale included in our patient information booklet.

During a face-to-face class, the patient’s response to exercise can be monitored via direct observation and heart rate and oxygen saturation measurements. Although fitness watches and monitors are available for public use, these monitoring methods are not always possible during virtual rehabilitation due to the cost implications for either the patient or the rehabilitation service. Patients are instead encouraged to self-monitor their levels of exertion using the Borg breathlessness scale, aiming for a moderate level of breathlessness, rated as 3 to 4 on the 12-point scale (51) (Figure 3). To maintain an appropriate level of breathlessness, patients should titrate their chosen exercise level throughout the class. Education and advice regarding breathlessness, the response to exercise and self-pacing are verbally discussed before each class. This information was also provided in writing via an electronic class information booklet, which was emailed to each patient after their pre-assessment and before starting the programme. The patients’ Borg breathlessness would be checked and documented at regular intervals during the class. The class therapist would share the image of the Borg scale on screen and ask patients to verbally report their current level of perceived breathlessness half-way through the class and at the end of the class. This was then reported in the patients records, along with their level of exercise intensity. If the patient’s level of breathlessness was too high or too low, the class therapist would advise on exercise titration accordingly. Patients were also regularly reminded to self-monitor their exercise intensity and breathlessness levels during the class and encouraged to speak to the class therapist if they had any queries or concerns.

### 3.7 Theme 7: privacy and information governance

In addition to health and safety measures, staff also expressed their concerns for information governance during remote service development, particularly in a group setting.

P10: “*We also need to think about information governance, especially if patients are in a public space or being overheard or watched by others. Especially in the support groups, patients need to feel they in a safe virtual space of trust.”*

The remote service was therefore planned to optimise patient information security and confidentiality. No concerns or complaints of information governance breach were reported during the service evaluation period. Recommendations are detailed below.

During virtually-delivered group exercises, patient privacy and information governance regulations should also be considered. The link to access the virtual group should be sent to each patient via a secure email account, with a new meeting link being shared each week, rather than having an open online forum. The class therapist should be the ‘host’ of the virtual meeting, ensuring to check the identity of the patient as they are permitted onto the call. In addition to a written and verbal disclaimer regarding exercise safety, privacy and confidentiality statements should also be provided before virtual group participation. This information should advise:

- Recordings of the class are not allowed.
- The meeting link must not be shared.
- The video call must only be joined when the confidentiality of all patients on the call can be maintained, i.e., they must not join in a public place where patients can be seen or overheard.
- To treat one another’s information with respect and confidentiality. If relatives are to join the call, they are to do the same.

Although information governance policies and procedures are understood and abided by staff, patients must also be informed of some of the essential guidance when attending group sessions. This privacy statement was necessary as our virtual exercise class was followed by a support group led by physiotherapists and critical care follow-up nurses. The support sessions involved group discussions about issues pertinent to critical illness recovery and advice, support, and education (38). The groups can be vastly heterogenous in terms of age, gender, ethnicity, cause for admission or health status, yet their commonality is their shared experience of critical illness and ICU admission. A virtual environment of trust and respect is necessary for patients to feel safe to share their personal experiences, which could often be private, emotional or distressing. Participation in the support group was voluntary and patients only engaged in conversation if they wished to do so. Patients were made aware that they could request for a member of the team to call them individually if they had a personal or private matter to discuss, such as a change in medical status or address. The information governance disclaimer aims to protect the privacy and confidentiality of all patients, providing a safe and personable virtual support group.

Furthermore, as part of routine training, it is mandatory that all staff complete Information Governance training. All staff have awareness of legislation such as the Data Protection Act (1998) in addition to local and national Information Governance policy.

With patient consent, the patient is preferred to always remain visible on the screen so the therapist can observe and monitor the patient throughout. However, the patient must be aware that all other patients in the group can also view them. In some circumstances, such as cultural preferences, the patient may choose to turn their camera off. The risks of not being visible should be explained to the patient so they can make an informed choice, and regular verbal feedback should be encouraged to check patient safety.

## 4 Discussion

The results of the multidisciplinary stakeholder focus groups identified seven critical themes for telerehabilitation service development. This innovative and agile response following the first wave of the COVID-19 pandemic informed the rapid development of an inclusive and successful post-intensive-care follow-up service. Our subsequent local service evaluation at one of the largest co-located ICUs in Europe demonstrated the safety, feasibility and effectiveness of virtual post-intensive-care rehabilitation for survivors of COVID-19 (34).

Telehealth, including telerehabilitation, has evolved rapidly since the COVID-19 pandemic and is now recommended as an alternative to face-to-face service delivery. In a post-pandemic era, rehabilitation services have to respond and react to modern healthcare challenges. Virtual adaptation may offer a logical, cost-effective and practical solution. With an ageing population and growing prevalence of multi-morbidity, there is an urgent need for equitable, inclusive and modernised rehabilitation (30, 31). Modernisation should consider the population’s changing needs, incorporating secondary prevention of complex long-term conditions, vocational rehabilitation and prehabilitation (30). This crisis presents an opportunity to create and implement accessible healthcare within the current National Health Service (NHS) constraints.

The constraints of modern-day therapy services lead to workforce stress and dissatisfaction and ultimately, deleterious effects on staff retention in an already under-resourced area (33). For the patient, there are significant consequences of delayed rehabilitation upon health-related outcomes (31). Across numerous sectors of outpatient and community rehabilitation, delayed therapy leads to devastating consequences upon pain, functional disability and both physical and psychological health-related quality of life (52, 53). Prolonged waiting times directly lead to poor health outcomes and increased healthcare utilisation and NHS costs (31, 53). Telehealth may be a solution during the post-pandemic recovery.

In the future, telehealth may be a solution for delivering accessible and cost-effective post-intensive-care follow-up services and other vital primary healthcare services (54–57). However, services must be inclusive, flexible and ensure to meet the needs and preferences of their service users. Where possible, patients should be provided with a choice of alternative services when virtual or group-based rehabilitation is not appropriate, such as patients with communication impairments, language barriers, learning difficulties or without technology access (44–46). Nonetheless, the common barriers to face-to-face follow-up in a post-pandemic era, such as lack of space, staff, resources and funding, can all be overcome with virtual rehabilitation (3). With over 5 billion internet users worldwide, reaching more than 66% of the global population, telehealth could centralise resources and reduce waiting list times worldwide (58).

### 4.1 Limitations

This report is based on the development of a single-centre rehabilitation service and local service evaluation study and thus has several limitations. Firstly, due to the time and logistical constraints of the pandemic, the stakeholder group consisted of staff members only and did not incorporate patient or public involvement. The group was established by convenience sampling methods, using existing staff members from the currently available services and thus may be subject to bias. The group may also be biased due to limited geographical, socio-demographic or ethnic diversity, however these factors were not recorded. Furthermore, as the researcher and facilitator was known to the focus-group members and a clinician heavily invested in the service development, findings may be further subject to bias and assumptions. The relationships and interactions between participants between each focus-group may have also confounded results.

As the original study was a single-centre service evaluation in the United Kingdom, for survivors of COVID-19 critical illness, without randomisation or a control group, we recognise that the results may lack validity and application to a wider audience. Further prospective trials are required to evaluate the generalisability and validity of such services beyond the pandemic and for survivors of all causes of critical illness. The guidance provided is based on best practice and clinical expertise at the time of service set-up, yet requires large-scale validation to confirm its wider utility.

## 5 Conclusion

This article shares the experiences and learning from the unique circumstances of the COVID-19 pandemic, requiring rapid and innovative virtual service development during the time of a global pandemic. We hope this may provide a blueprint for others looking to set up similar services, providing practical advice and support to empower clinicians to set up and deliver virtual post-intensive-care rehabilitation and follow-up services. Virtual adaptations offer a pragmatic, accessible, and cost-effective alternative to rehabilitation, where resources are scarce and under-provided nationally. Virtual rehabilitation is safe, feasible, and supported by national guidelines, but it needs appropriate resources, time investment, and careful planning. Patient safety and risk assessment should be at the forefront of development, ensuring processes are in place to mitigate risk. Although a logical alternative in a post-pandemic era, further research is required to evaluate the efficacy of virtual services and patient experiences.

## Data Availability

The raw data supporting the conclusions of this article will be made available by the authors, without undue reservation.
